# The New Hospital in Shillong, Assam

**Published:** 1920-10-30

**Authors:** 


### The New Hospital in Shillong, Assam.

A MISSION DOCTOR'S ENTERPRISE.

An exhibition of Oriental life has been held at Cardiff in aid of the new hospital in Shillong, Assam, the foundation of which is due to the self-sacrifice of Dr. H. Gordon Roberts, a doctor who has devoted himself to Mission work. A Welshman by birth, after qualification he decided to enter the Mission-field in India, whither he went some seven years ago. The condition of Shillong, Assam, seemed to him so appalling that he settled there, determined to do something to supply the lack of hospital accommodation and medical treatment in his district of a quarter of a million people.

During the war the Government appropriated his services, and for a long time he was the one European doctor in Shillong. His services were rewarded by an honorarium of £3,000 from the Government, a sum which he handed over to the funds of the new hospital, while he contented himself with his salary of £200 a year as a Mission doctor. This act of self-sacrifice induced the Government to subscribe £4,000 to the hospital's funds. So the foundation of the institution was secured with £7,000.

To obtain the rest of the money required Dr. Roberts is visiting this country. The exhibition was carefully planned to illustrate those sides of Oriental life about which the public is curious—the Zenana and the opium den, for instance—and there was also a medical section to emphasise the contrast between Eastern and Western methods, and lectures were given by qualified doctors and nurses with experience of the work. Oriental plays were also performed. These show the enthusiasm with which the object is being pursued, and give us one of the too rare glimpses of the interests and ambitions of the doctor in the Mission-field.

